# An enhanced Transformer framework with incremental learning for online stock price prediction

**DOI:** 10.1371/journal.pone.0316955

**Published:** 2025-01-13

**Authors:** Yiming Qian

**Affiliations:** Harvard extension school, Harvard University, Boston, Massachusetts, United States of America; Virtual University of Pakistan, PAKISTAN

## Abstract

To address the limitations of existing stock price prediction models in handling real-time data streams—such as poor scalability, declining predictive performance due to dynamic changes in data distribution, and difficulties in accurately forecasting non-stationary stock prices—this paper proposes an incremental learning-based enhanced Transformer framework (IL-ETransformer) for online stock price prediction. This method leverages a multi-head self-attention mechanism to deeply explore the complex temporal dependencies between stock prices and feature factors. Additionally, a continual normalization mechanism is employed to stabilize the data stream, enhancing the model’s adaptability to dynamic changes. To ensure that the model retains prior knowledge while integrating new information, a time series elastic weight consolidation (TSEWC) algorithm is introduced to enable efficient incremental training with incoming data. Experiments conducted on five publicly available datasets demonstrate that the proposed method not only effectively captures the temporal information in the data but also fully exploits the correlations among multi-dimensional features, significantly improving stock price prediction accuracy. Notably, the method shows robust performance in coping with non-stationary and frequently changing financial market data.

## 1. Introduction

With the rapid development of market economies, the stock market has expanded significantly, attracting a growing number of investors. Accurate prediction of stock price trends not only aids in optimizing investment decisions but also substantially enhances returns [1, 2]. However, stock prices, as a typical financial time series, are influenced by numerous factors and exhibit characteristics such as high dimensionality, non-stationarity, and stochastic volatility. These features make precise forecasting particularly challenging [3, 4].

Traditional stock price prediction methods, such as autoregressive integrated moving average (ARIMA) models, rely on historical information to make linear predictions of future values [5, 6]. However, as big data and deep learning technologies have advanced, hybrid models combining statistical approaches with machine learning and neural networks have gained prominence [7]. These models effectively address the non-linear relationships and large-scale data inherent in stock price prediction, achieving significant improvements in forecasting accuracy and directional prediction. Despite these advancements, existing methods face limitations in modeling local and long-term dependencies in time series data, maintaining sensitivity to trend changes, and overcoming computational complexity or issues such as gradient vanishing or explosion [8].

In contrast, Transformer models, known for their strong global information extraction capabilities, have emerged as a promising approach for time series analysis. Initially developed for sequence-to-sequence tasks such as natural language translation, Transformers excel at modeling both short-term and long-term dependencies, demonstrating superior performance in various applications [9, 10]. However, most existing studies on Transformers employ batch learning paradigms that rely on complete historical data and assume a static relationship between inputs and outputs. This static assumption fails to account for the dynamic nature of data distribution over time. When new data arrive incrementally, batch retraining becomes computationally expensive and impractical, especially under data privacy constraints where access to complete historical data may be restricted. Incremental learning, therefore, is critical for addressing these challenges in real-time data scenarios.

Incremental learning holds significant potential for adapting to dynamic data distributions but often encounters the problem of catastrophic forgetting, where learning new data severely degrades the model’s performance on previously learned data. This challenge, known as the stability-plasticity dilemma, involves balancing the retention of old knowledge with the efficient acquisition of new knowledge. To address this issue, three primary paradigms of incremental learning have been explored: regularization, replay, and parameter isolation. For instance, Elastic Weight Consolidation (EWC) is a representative regularization-based method [11], Experience Replay (ER) [12] and its variant DER++ [13] employ replay mechanisms, and PackNet [14] exemplifies parameter isolation strategies. However, most incremental learning research focuses on class-incremental tasks, while regression tasks, such as stock price prediction, remain underexplored and warrant further investigation.

Based on the aforementioned challenges, this study proposes an incremental online stock price prediction model, IL-ETransformer, to address the forecasting of non-stationary stock price sequences. To handle the complex temporal dependencies inherent in stock price data, the model integrates a multi-head attention mechanism. Unlike traditional time series modeling methods, which struggle to simultaneously capture both short-term local features and long-term global dependencies, the multi-head attention mechanism leverages parallel attention heads to extract diverse temporal patterns from the same input sequence. Given the pronounced non-stationarity of stock price sequences, direct modeling may lead to unstable predictions or cumulative bias. To mitigate this, the model incorporates a continuous normalization mechanism, dynamically stabilizing the data stream. This mechanism reduces prediction errors caused by distributional shifts, enhances the adaptability of the model during incremental learning, and ensures consistent predictive performance in real-time environments. Additionally, to address the common challenge of catastrophic forgetting in incremental learning, the study employs a time series elastic weight consolidation (TSEWC) algorithm to facilitate incremental training. By dynamically adjusting and balancing weights, the model effectively retains knowledge of historical data while learning new information, achieving an optimal balance between stability and plasticity. The contributions of this study are summarized as follows:

- An IL-ETransformer model is developed for dynamic online stock price prediction, effectively maintaining high predictive accuracy within an incremental learning framework. This approach offers a novel solution for forecasting non-stationary time series data.
- The incorporation of a multi-head attention mechanism enhances the model’s ability to capture multi-scale features, enabling a more comprehensive exploration of complex temporal dependencies between stock prices and feature factors. Additionally, a continual normalization mechanism was employed to dynamically stabilize the data stream, significantly improving the model’s adaptability to dynamic changes in time series data.
- Building upon the traditional EWC algorithm, a TS-EWC algorithm is proposed, specifically optimized for stock price prediction tasks. This method is more suitable for addressing dynamic incremental learning challenges in financial time series data and effectively mitigates the issue of catastrophic forgetting.

The remainder of this paper is organized as follows: Section 2 reviews related work; Section 3 presents the foundational theory of the proposed algorithm; Section 4 elaborates on the algorithm’s design details; Section 5 evaluates its predictive performance; and Section 6 concludes with a summary of findings.

## 2. Related work

### 2.1 Current research on stock price prediction

With the rapid development of the big data era, deep learning technologies have made breakthrough progress due to their powerful learning and feature extraction capabilities. Three commonly used deep learning models for time series prediction are Convolutional Neural Networks (CNN), Recurrent Neural Networks (RNN), and Long Short-Term Memory Networks (LSTM). However, these models face challenges such as high computational complexity and limited sequence utilization. Qiu et al. decomposed stock features and applied an RNN model for stock price prediction, demonstrating the effectiveness of RNNs in the stock market [15]. LSTM, a variant of RNN, introduces gate mechanisms that effectively address the gradient vanishing and explosion problems that occur during the training of long sequences in RNNs. Mehtab et al. enhanced LSTM using forward validation methods and optimized the model’s hyperparameters through grid search [16]. Yu et al. integrated time-series phase space reconstruction algorithms into LSTM models for stock price prediction [17]. Thakkar et al. reviewed the state of research on deep learning in stock prediction and discussed future directions [18]. Lu et al. developed a CNN-LSTM model for stock price prediction, where CNN is used for feature extraction and LSTM for prediction [19]. Zhang et al. proposed a hybrid model combining CNN, LSTM, and attention mechanisms, which performed well in stock market price prediction [3]. Lu et al. introduced a method that combines CNN for feature extraction with BiLSTM to predict stock prices, providing reliable reference for investors’ decisions [20]. Chung et al. addressed the labor-intensive issue of manually tuning CNN hyperparameters by introducing a genetic optimization algorithm for automatic parameter selection [21]. However, these conventional deep learning methods still exhibit limitations in time series data modeling, including insufficient capture of local and long-term dependencies, low sensitivity to trend changes, and bottlenecks in computational complexity and gradient vanishing or explosion issues.

The Transformer model has demonstrated significant advantages in stock price prediction due to its powerful global modeling ability, efficient parallel computation architecture, flexible feature extraction mechanism, and adaptability to dynamic data changes. It has attracted significant attention in the field of stock price prediction. Ding et al. combined the Transformer model with a hierarchical multi-scale Gaussian mixture model, achieving good performance in capturing both long-term and short-term dependencies in stock price data [22]. Li et al. preprocessed raw stock price data using an empirical model and constructed a hybrid model that integrates Gated Recurrent Units (GRU), LSTM, and Transformer for predictive analysis of the preprocessed data [23]. Wang et al. designed a new Transformer model, MTRAN-TCN, based on BiLSTM, Temporal Convolutional Networks (TCN), and Transformer, and validated its effectiveness with 19 stocks [24]. Li et al. incorporated market sentiment and volatility factors into a generative adversarial network (GAN) to generate data, and combined this with Transformer models for stock price prediction [25]. Experimental results showed that this method effectively integrated various external factors, significantly improving prediction accuracy and robustness. Zhang et al. introduced multiple attention mechanisms into the Transformer, achieving precise stock price prediction with limited sample data [26].

Despite the exceptional performance of current Transformer models in stock price prediction tasks, their strong global information modeling ability allows them to capture complex time series features. However, these models typically rely on full historical data for training, assuming that the relationship between input and output is static and does not change over time. This assumption often fails in real-world applications, as stock price distributions dynamically adjust with market conditions and external factors. Additionally, when new data arrives, traditional methods require retraining the model, which is time-consuming and computationally expensive. Moreover, data privacy and security restrictions may hinder the availability of complete historical data. Therefore, the introduction of incremental learning becomes a necessary approach to address these issues. Incremental learning enables the model to gradually update as new data arrives, allowing it to adapt to dynamic data distributions while avoiding dependence on complete historical data. This improves the real-time applicability of the model and meets the complex demands of stock price prediction in practical applications.

### 2.2 Current research on incremental learning

Incremental learning is a machine learning approach designed to continuously learn new knowledge without forgetting existing knowledge. The core objective is to enable a model to adapt to new information by adjusting its parameters or updating its structure when new data or tasks are introduced, while retaining knowledge from previous tasks. Incremental learning has significant advantages in handling continuous streaming data, as it satisfies the requirements for real-time processing and dynamic adaptation, making it particularly suitable for scenarios where data distributions change over time. In recent years, significant progress has been made in the application of incremental learning to time series data prediction.

In the area of ensemble learning, Wang et al. proposed an incremental training method based on extreme learning machine (ELM), which achieved online ensemble learning [27]. Building upon this, Yu et al. introduced a dynamic weight update ensemble learning algorithm (DWE-IL), which significantly improved the model’s generalization performance by dynamically adjusting the parameters of the base model ELMK (Extreme Learning Machine with Kernels) [28]. However, as time progresses, the differences between the base models in the ensemble can gradually increase, leading to the accumulation of prediction errors. In the field of deep learning models, researchers have developed various incremental models suitable for online learning by optimizing the network structure. Wang et al. proposed the IncLSTM (Incremental Ensemble LSTM Model), which optimized the LSTM structure for incremental time series prediction [29]. Woo et al. improved the Temporal Convolutional Network (TCN) by proposing the Online-TCN model, which maintains high prediction accuracy for time series data while exhibiting efficient incremental updating capabilities [30]. Additionally, Huang et al. optimized the Transformer model, incorporating Bayesian estimation to design an incremental Transformer model more suitable for stream data computation [31].

Regarding specific incremental learning algorithms, EWC is one of the classical methods. This algorithm evaluates the importance of each parameter in a neural network and penalizes changes in crucial parameters during subsequent task training to retain knowledge from previous tasks. However, traditional EWC algorithms have certain limitations when dealing with complex nonlinear time series data, such as their inability to adequately adapt to dynamically changing data distributions. To address this, the present study introduces the EWC algorithm into stock price prediction tasks and improves it to develop an incremental online stock price prediction model, IL-ETransformer. This model optimizes the EWC algorithm to better handle dynamic financial time series data while integrating the Transformer’s multi-head attention mechanism and continuous normalization mechanism, enhancing its ability to process non-stationary stock price data. This provides new insights and solutions for the application of incremental learning in the financial domain.

## 3. Theoretical foundation

### 3.1 Online time series prediction

Let $\chi \text{=}(x_{1},x_{2},\cdots ,x_{L})\in {\text{ℝ}}^{L\times n}$ be a time series of length *L*, where each observation *x*_*i*_ has a dimension of *n*, represented as a token. The objective of time series prediction is to forecast the future *H*-step time series, given a lookback window *χ*_*i*,*e*_ = (*x*_*i*−*e*+1_, *x*_*i*−*e*+2_, ⋯,*x*_*i*_), aiming to minimize the discrepancy between predicted values and actual values. Specifically, this task can be formulated as:

$$f_{\omega }(\chi_{i,L})=(x_{i+1},x_{i+2},\cdots ,x_{i+H})$$ (1)

where *ω* denotes the parameters of the prediction model.

In realistic settings, particularly in financial time series characterized by non-stationarity, high complexity, and random fluctuations, it is more appropriate for data to arrive sequentially in a stream. For online time series prediction tasks, the learning process occurs over multiple rounds. In each round, the model receives a lookback window, makes predictions for the forecast window, and then updates the model using the revealed true values to improve predictions for future rounds. This enables the model to learn new knowledge from continuous input streams, alternating between training and prediction.

### 3.2 Elastic weight consolidation for incremental learning

The EWC algorithm is designed to selectively regularize network parameters in an incremental learning setting. It leverages the Fisher Information Matrix to identify crucial directions in the parameter space that are vital for previously learned tasks. By constraining the most important parameters to retain prior knowledge, EWC reduces the learning rate for weights that are essential for old tasks, thus mitigating catastrophic forgetting.

Based on Bayesian theory, given a dataset *D*, the conditional probability *p*(*θ*|*D*) can be derived from the prior probability of the parameters *p*(*θ*) and the likelihood of the data *p*(*D*|*θ*), expressed as:

$${\text{log}}_{a}\;p(\left.\theta \right|D)={\text{log}}_{a}\;p(\left.D\right|\theta )+{\text{log}}_{a}\;p(\theta )-{\text{log}}_{a}\;p(D)$$ (2)

If the data is given, the log-likelihood can be defined as the negative loss function. Assuming that the data is divided into two independent parts, denoted as *D*_*A*_ and *D*_*B*_, the formula can be modified as follows:

$${\text{log}}_{a}\;p(\left.\theta \right|D)={\text{log}}_{a}\;p(\left.D_{B}\right|\theta )+{\text{log}}_{a}\;p(\theta \left|D_{A}\right.)-{\text{log}}_{a}\;p(D_{B})$$ (3)

On the left-hand side of Eq (3), the posterior probability given the entire dataset is described, while on the right-hand side, it depends solely on the loss function for task *B*. All information from task *A* is embedded within the posterior distribution *p*(*θ*|*D*_*A*_). Since the posterior distribution is difficult to compute directly, it can be approximated using a Gaussian distribution with the diagonal elements of the Fisher information matrix, which provides the parameter $\theta_{A}^{*}$ and the precision of the posterior approximation. The loss function *L*(*θ*) in EWC can then be minimized:

$$L(\theta )=L_{B}(\theta )+\sum_{i}\frac{\lambda }{2}F_{i}{\left(\theta_{i}-\theta_{A,i}^{*}\right)}^{2}$$ (4)

Where *L*_*B*_(*θ*) is the loss function for task *B*, and *λ* denotes the importance of each parameter *i* in preserving knowledge from previous tasks.

## 4. Reinforced transformer framework with incremental learning

**Fig 1:**
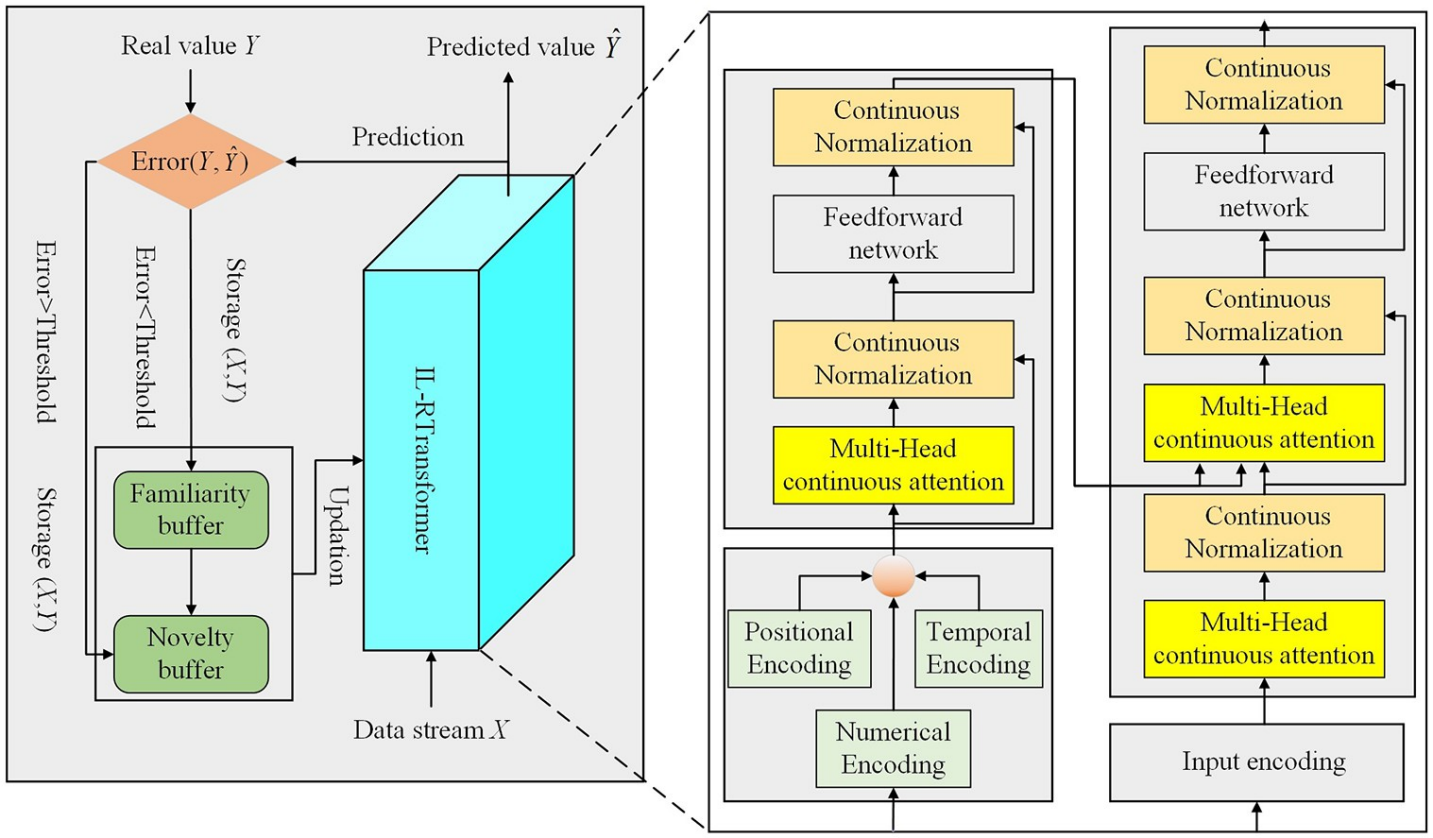
The model structure of proposed IL-ETransformer framework.

IL-ETransformer is a time series prediction framework that integrates deep learning with incremental learning. The model is built upon the Transformer architecture, using an input encoding module to extract temporal features, generating input vectors for the encoder. Both the encoder and decoder utilize a continual attention mechanism, which is more suitable for incremental learning scenarios, while a continual normalization mechanism replaces the standard layer normalization. The encoder captures features from the input time series, and the decoder uses these extracted features for time series prediction, passing them through a linear fully connected layer to produce the final output. Moreover, the training framework updates the model using an enhanced time series elastic weight consolidation algorithm (TS-EWC), enabling the model to continually learn new knowledge and improve prediction accuracy. The overall architecture of the IL-ETransformer for stock price prediction is shown in Fig 1.

### 4.1 Input encoding

The input encoding in the model follows a setup similar to that in Informer [32], consisting of three components: data embedding, position embedding, and timestamp embedding. A one-dimensional convolutional network with a kernel size of 3 projects the input sequence *X*_*in*_, originally in the *d*_*in*_-dimensional input space, to a *d*_model_ -dimensional model space to align the data dimensions with the model, producing the data embedding vector. Additionally, position embeddings for *X*_*in*_ are incorporated to capture the relative positional information of the input sequence, providing sequential features for the input. Since the timestamp corresponding to *X*_*in*_ is also part of the input features, timestamp embedding is introduced to capture the global temporal characteristics of the input.

The timestamp of the price series includes information such as year, month, day, and day of the week, which is sequentially encoded and merged. After combining the data embedding, position embedding, and timestamp embedding, the final input encoding vector is obtained.


$$X_{\text{emb}}=data_{\text{emb}}+pos_{\text{emb}}+time_{\text{emb}}$$ (5)

### 4.2 Encoder and decoder

The IL-ETransformer encoder is composed of multiple identical layers, each containing three sub-layers: continual multi-head attention (ConMHA), feed-forward network (FFN), and continual normalization (CN). After the raw input sequence *X*_*in*_ is processed by the input encoding module, it becomes the input sequence *X*_emb_ for the encoder.

The IL-ETransformer decoder follows a similar structure to the encoder, with the addition of a masked continual multi-head attention module in its sub-layers. This module assigns different weights to the intermediate result *Q* of the decoder, based on the encoder outputs *K* and *V*, to compute the relevance between *K* and *Q*. Continual normalization is also applied to process data between the network sub-layers within the decoder. The input vector for the decoder is as follows:

$$X_{\text{de}}=Concat(X_{\text{token}},X_{0})$$ (6)

where *X*_token_ is the initial input of the historical sequence with length *T*_token_, and *X*_0_ is a placeholder for the predicted sequence, also with length *H*, filled with zeros. *Concat*() denotes the concatenation operation. After being fed into the decoder, the input passes through a series of network layers and is finally processed by a linear fully connected layer to adjust the output length, resulting in the final prediction.

### 4.3 Continual multi-head attention

The self-attention mechanism in Transformer models captures the similarity relationships between data, identifying and preserving the importance of connections between elements. This mechanism follows a query-key-value (*QKV*) model to compute the similarity between each element and others, thereby extracting temporal dependencies. The calculation is described as:

$$Att(Q,K,V)=D^{-1}AV$$ (7)

$$A=\text{exp}(QK^{\text{T}}/\sqrt{d}),D=diag(A\cdot I_{L})$$ (8)

where $Q\in {\text{ℝ}}^{L\times d_{\text{model}}}$, $K\in {\text{ℝ}}^{L\times d_{\text{model}}}$, $V\in {\text{ℝ}}^{L\times d_{\text{model}}}$, and *d*_model_ represent the dimensions of the hidden layers, and $I_{L}$ is a column vector with all elements equal to 1. The exponential function exp(⋅) is applied element-wise. The model’s time complexity is $O(nL^{2})$, and its space complexity is $O(L^{2})$, where *L* and *n* denote the length and dimensionality of the time series, respectively.

Considering online time series prediction scenarios, IL-ETransformer updates continuously by processing data incrementally as it arrives sequentially. At each time step, the earliest token is discarded, and a new token is added in a first-in-first-out manner to update *Q*, *K*, and *V*. In this context, attention scores can be efficiently recalculated using cached historical results and the most recent queries, keys, and values $\left(q_{new},k_{new},v_{new}\in {\text{ℝ}}^{1\times n}\right)$, enabling effective updates of *D*^−1^ through matrix multiplication. By caching the previous *L*−1 values, denoted as $d_{mem}=A_{pre}^{\left(2:L\right)}I_{L-1}$, the update process can be described as:

$$d^{\left(1:L-1\right)}=d_{mem}^{\left(2:L\right)}-\text{exp}\left(\frac{Q_{mem}k_{old}^{T}}{\sqrt{d_{\text{model}}}}\right)+\text{exp}\left(\frac{Q_{mem}k_{new}^{T}}{\sqrt{d_{\text{model}}}}\right)$$ (9)

$$d^{L}=\text{exp}\left(q_{new}\frac{Concat(K_{mem},k_{new})}{\sqrt{d_{\text{model}}}}\right)I_{L}$$ (10)

Where *Q*_*mem*_ and *K*_*mem*_ represent the cached queries and keys from the previous *L*−1 tokens, and *k*_*old*_ represents the token keys from the previous *L* time steps. The attention values *AV* can also be updated based on the prior *AV*_*mem*_ as:

$$AV^{\left(1:L-1\right)}=AV_{mem}^{\left(2:L\right)}-\text{exp}\left(\frac{Q_{mem}k_{old}^{T}}{\sqrt{d_{\text{model}}}}\right)v_{old}+\text{exp}\left(\frac{Q_{mem}k_{new}^{T}}{\sqrt{d_{\text{model}}}}\right)v_{new}$$ (11)

$$AV^{\left(L\right)}=\text{exp}\left(q_{new}\frac{Concat(K_{mem},k_{new})}{\sqrt{d_{\text{model}}}}\right)Concat(V_{mem},v_{new})$$ (12)

The output of the continual attention mechanism is described as:

$$ConAtt(q_{new},k_{new},v_{new})=d^{-1}⊙AV$$ (13)

This improved continual attention mechanism reduces the time and space complexity per time step to O(nL), making it more suitable for streaming computations and significantly lowering computational overhead.

In practice, a multi-head attention mechanism is employed to project inputs into multiple subspaces using different sets of *Q*, *K*, and *V*, which enhances the model’s ability to capture dependencies across various dimensions of the sequence. Given a new set of query-key-value pairs, the continual multi-head attention (ConMHA) can be described as:

$$ConMHA(q,k,v)=Concat(head_{1},head_{2},\cdots ,head_{h})W_{o}$$ (14)

$$head_{i}=ConAtt(qW_{Q}^{i},kW_{K}^{i},vW_{V}^{i})$$ (15)

where $W_{Q}^{i}\in {\text{ℝ}}^{d_{\text{model}}\times d_{k/h}}$,$W_{K}^{i}\in {\text{ℝ}}^{d_{\text{model}}\times d_{k/h}}$,$W_{K}^{i}\in {\text{ℝ}}^{d_{\text{model}}\times d_{k/h}}$ and $W_{o}\in {\text{ℝ}}^{d_{v}\times d_{\text{model}}}$.

In an online learning setting, when new data arrives at time step *t*+1, the model updates its parameters based on historical data while generating predictions for the current time step. Through the use of parallel heads, the ConMHA mechanism models weighted summations of diverse features, enabling it to effectively capture emerging patterns even as data distributions evolve. Additionally, the weights of individual attention heads are dynamically adjusted to accommodate these changes. Importantly, the incremental nature of parameter updates ensures that the model remains responsive to variations in the input data stream.

### 4.4 Continual normalization mechanism

In the Transformer model, layer normalization (LN) is designed to handle variable-length sequential data by normalizing across the features of individual samples, considering only the sample dimension. Group normalization (GN) [33], on the other hand, normalizes groups of features within each sample, where LN can be seen as GN with a group size of 1. Batch normalization (BN) [34] normalizes based on the same features across different samples, facilitating faster convergence during network optimization. The standard normalization formula is:

$$y=\gamma \times \frac{x-\mu }{\sqrt{\sigma^{2}+\varepsilon }}+\beta$$ (16)

Where *μ* and *σ*^2^ represent the mean and variance of the input features, *ε* is a small constant to avoid division by zero, and *γ* and *β* are affine transformation parameters learned during training, with initial values *γ* = 1 and *β* = 0. The specific range of the affine-transformed data depends on the dataset. The computation of the mean and variance for BN and GN follows Eqs (17) to (19).


$$\mu_{BN}=\frac{1}{BHW}\sum_{b=1}^{B}\sum_{w=1}^{W}\sum_{h=1}^{H}x_{bcwh},\sigma_{BN}^{2}=\frac{1}{BHW}\sum_{b=1}^{B}\sum_{w=1}^{W}\sum_{h=1}^{H}(x_{bcwh}-\mu_{BN}{)}^{2}$$ (17)

$$x_{bgkwh}^{\prime }\leftarrow x_{bcwh},k=\left\lfloor *\right\rfloor \frac{C}{G}$$ (18)

$$\mu_{GN}^{\left(g\right)}=\frac{1}{m}\sum_{k=1}^{K}\sum_{w=1}^{W}\sum_{h=1}^{H}x_{bcwh}^{\prime },\sigma_{GN}^{2}=\frac{1}{m}\sum_{k=1}^{K}\sum_{w=1}^{W}\sum_{h=1}^{H}(x_{bcwh}^{\prime }-\mu_{GN}^{\left(g\right)}{)}^{2}$$ (19)

For time series tasks, BN tends to disrupt the temporal dimension’s dependencies. To combine the strengths of GN and BN, a continual normalization (CN) mechanism is introduced, which is more suited for incremental data scenarios. It first performs spatial normalization using GN on the feature maps, followed by further feature normalization using BN. The computation for CN is described as:

$$x_{GN}\leftarrow GN_{1,0}(x);x_{CN}\leftarrow \gamma BN_{1,0}(x_{GN})+\beta$$ (20)

In incremental learning settings, where data arrives sequentially, CN replaces the batch mean and variance in Eq (17) with estimated global values from the training process:

$$\mu \leftarrow \mu +\eta (\mu_{B}-\mu ),\sigma \leftarrow \sigma +\eta (\sigma_{B}-\sigma )$$ (21)

In an incremental learning environment, normalization ensures that the scale of input data remains consistent during each model update. This consistency mitigates the instability caused by the non-stationarity of the input data.

### 4.5 Time series elastic weight consolidation for incremental learning

In the IL-ETransformer framework, model updates and trigger conditions play a crucial role. Stock price data often exhibit non-stationarity, necessitating that the model incrementally learn from new data when the distribution changes. For online time series prediction tasks, an improved version of the EWC algorithm, TS-EWC algorithm is proposed.

Each time the data distribution changes, new data is treated as a new task for the model to learn. Over time, EWC maintains a penalty term for each historical task generated from the data stream. As the number of tasks increases, the number of penalty terms grows linearly, resulting in significant computational costs. However, since each new task imposes a penalty term based on the previous task, the model update only needs to maintain the most recent penalty term, and the Fisher matrix for previous tasks is weighted and summed. The loss function in TS-EWC is defined as:

$$L(\theta )=L_{D}(\theta )+\sum_{i}\frac{\lambda }{2}\sum_{d<D}\lambda_{d}F_{t,i}{\left(\theta_{i}-\theta_{D-1,i}^{*}\right)}^{2}$$ (22)

Where *L*_*D*_(*θ*) represents the loss for the current task, and the model update trigger is defined by a hyperparameter. Two buffers are set: one to measure mean square error as a threshold, comparing it with incoming data to detect novelty or familiarity. The novelty buffer monitors changes in the data stream’s probability distribution, triggering model updates when necessary, and dynamically adjusting the threshold post-update. The familiarity buffer contains previously learned information, ensuring that, after updating, the model retains prior knowledge and can quickly predict recurring patterns in future data streams.

In the traditional EWC algorithm, the importance metric *F*_*i*_ is estimated using the Fisher information matrix, which quantifies the influence of parameter *θ*_*i*_ on the model’s output. During incremental learning, the Fisher information matrix is updated as new data becomes available. The improved EWC algorithm enhances adaptability to financial time series data by dynamically adjusting *F*_*i*_, thereby improving the model’s ability to accommodate changes in data distribution. This adaptation enables the model to more effectively handle the time-varying characteristics of financial data.

## 5. Experimental results and analysis

### 5.1 Dataset and experimental setup

The experiments were conducted to predict both index and individual stock prices. Data were obtained from the iFinD financial database [35], which provides daily trading data for China’s stock market. Specifically, the datasets include the Shanghai Shenzhen 300 Index (000300.SH) and the China Securities 500 Index (399905.SZ) for the period from January 16, 2007, to December 29, 2023. Given the vast number of stocks in the market, representative stocks from three prominent industries—finance, media, and pharmaceuticals—were selected from the component stocks of the two indices to enhance the generalizability and objectivity of the analysis. The chosen stocks are Ping An Bank, China South Publishing & Media, and Sinopharm. Daily trading data for these stocks were collected from January 4, 2010, to December 29, 2023. Each dataset includes variables such as moving average convergence divergence, change ratio, opening price, closing price, highest price, and lowest price. Table 1 provides a summary of the data and their statistical characteristics.

**Table 1:** Partial data display of 000300.SH.

Date	High	Low	Open	Close
2007-01-16	2354.43	2297.24	2310.96	2353.87
2007-01-17	2393.22	2266.34	2360.41	2308.93
—	—	—	—	—
2023-12-28	3423.40	3331.21	3335.56	3414.54
2023-12-29	3432.54	3409.58	3411.11	3431.11
mean	3473.68	3505.68	3441.08	3476.22
std	876.13	881.74	866.89	875.33
median	3437.49	3466.57	3413.22	3440.97
min	1614.62	1648.45	1606.73	1627.76
max	5922.07	5930.91	5815.61	5877.20

To eliminate the impact of different feature dimensions within the data, the sequences were normalized. The dataset was divided into three phases: warm-up, validation, and online prediction, with a ratio of 2:1:7. For the index data, the training set contains 845 samples, the validation set contains 422 samples, and the test set contains 2,956 samples. For the individual stock data, the training set contains 682 samples, the validation set contains 341 samples, and the test set contains 2,386 samples. During the warm-up and validation phases, the model was pre-trained, while in the online prediction phase, the dataset was sequentially fed into the model to simulate an ever-growing data stream. The look-back window was set to 60, and the prediction window was configured with a forecast horizon of 24. After each prediction, the true values were compared with the predicted values. The experimental model was built and computed using an Nvidia Tesla T4 GPU. The IL-ETransformer consisted of *N* = 2 encoders and *M* = 1 decoder. The Adam optimizer with a learning rate of 0.0001 was used during the warm-up phase, with mean squared error (MSE) as the loss function. In the online prediction phase, the TS-EWC algorithm was used to compute the model’s loss function and perform incremental updates.

### 5.2 Evaluation metrics

To provide a comprehensive assessment of the predictive performance of the IL-ETransformer model, four regression metrics were utilized: mean absolute error (MAE), MSE, root mean square error (RMSE), and mean absolute percentage error (MAPE). These metrics were employed to evaluate the prediction results, and their formulas are as follows:

$$MAE=\frac{1}{N}\sum_{n=1}^{N}\left|{\hat{y}}_{n}-y_{n}\right|$$ (23)

$$MSE=\frac{1}{N}\sum_{n=1}^{N}{\left({\hat{y}}_{n}-y_{n}\right)}^{2}$$ (24)

$$RMSE=\sqrt{\frac{1}{N}\sum_{n=1}^{N}{\left({\hat{y}}_{n}-y_{n}\right)}^{2}}$$ (25)

$$MAPE=\frac{1}{N}\sum_{n=1}^{N}\frac{\left|{\hat{y}}_{n}-y_{n}\right|}{y_{n}}$$ (26)

where *n* represents the total number of samples, *y*_*n*_ denotes the actual values, and ${\hat{y}}_{n}$ represents the predicted values. The smaller the values of the regression evaluation metrics—MAE, MSE, RMSE, and MAPE—the closer the predicted values are to the actual values, indicating higher prediction accuracy.

### 5.3 Experimental results and analysis

#### 5.3.1 Index prediction analysis

To evaluate the performance of the IL-RTransformer prediction framework, comparative experiments were conducted using various models from the fields of deep learning and incremental learning for stock price prediction: LSTM [16], Transformer [4], Informer [32], IncLSTM [29], DWE-IL [28], Online TCN [30], ER [12], and DER++ [11]. Among these, LSTM, Transformer, and Informer were implemented using an offline batch processing setup and trained exclusively during the preheating phase on the given dataset. Specifically, the training process for these models utilized mean squared error (MSE) as the loss function, the Adam optimizer, and a batch size of 64. For LSTM, the number of hidden units was set to 50, with ReLU as the activation function. Transformer and Informer employed layer normalization (LN) for network normalization, utilizing LogSparse attention and ProbSparse attention mechanisms, respectively. In contrast, IncLSTM, DWE-IL, and OnlineTCN were designed for incremental learning and updated dynamically during the online prediction phase as new ground truth data became available, enabling these models to adapt to evolving data patterns. IncLSTM adopted LSTM as the base model and incorporated the Tradaboost method for ensemble learning, with 50 iterations and a Tradaboost number set to 2. DWE-IL employed ELMK as the base model with an RBF kernel function and a review window length of 60. OnlineTCN was based on a standard TCN backbone, consisting of 10 hidden layers, each with two residual convolutional filters. Additionally, ER and DER++ employed Transformer as the backbone network for online prediction, maintaining the same buffer size as IL-RTransformer to revisit old samples. To ensure fair comparisons, all datasets were preprocessed using identical normalization techniques prior to model training. However, since Transformer-based models leverage affine transformations in their normalization mechanisms, these parameters were learned within the network layers. For consistency, affine transformations based on Transformer parameters were applied to the prediction results of other models, such as LSTM, ELM, and TCN, before comparison. Experiments were conducted using the CSI 300 Index and the CSI 500 Index datasets. The average values of evaluation metrics from 20 independent prediction runs were used for performance comparison. The prediction results of the models during the online prediction phase are presented in Table 2.

**Table 2:** Prediction results of 000300.SH and 399905.SZ.

Index	Model	MAE	MSE	RMSE	MAPE
000300.SH	LSTM	0.426	0.981	0.678	1.789
	Transformer	0.402	0.879	0.729	1.672
	Informer	0.322	0.712	0.742	1.644
	IncLSTM	0.362	0.763	0.678	1.498
	DWE-IL	0.331	0.542	0.615	1.484
	OnlineTCN	0.334	0.533	0.607	1.498
	ER	0.259	0.325	0.442	1.178
	DER++	0.229	0.295	0.427	0.899
	IL-ETransformer	**0.225**	**0.274**	**0.431**	**0.742**
399905.SZ	LSTM	0.415	0.622	0.718	1.160
	Transformer	0.379	0.451	0.696	1.158
	Informer	0.356	0.456	0.600	1.017
	IncLSTM	0.332	0.339	0.519	0.967
	DWE-IL	0.338	0.355	0.530	0.912
	OnlineTCN	0.327	0.346	0.510	0.989
	ER	0.251	0.235	0.401	0.728
	DER++	0.233	0.234	0.395	0.667
	IL-ETransformer	**0.228**	**0.215**	**0.384**	**0.637**

As shown in Table 2, the evaluation metrics for LSTM, Transformer, and Informer are notably higher. While LSTM, as a classic neural network model, effectively addresses the issues of vanishing and exploding gradients during the training process for time series data prediction, both Transformer and Informer leverage self-attention mechanisms to extract temporal information and reduce prediction error. However, these three models exhibit poor performance in the online prediction scenario. This is because they only utilize offline batch processing during the warm-up phase, and as the volume of prediction data increases, the models fail to accurately predict the new data distribution. For highly volatile and non-stationary time series data, such as stock prices, the prediction performance significantly deteriorates when the amount of prediction data exceeds the training data. IncLSTM, an incremental ensemble model based on LSTM, continuously learns new data during the online prediction phase and employs multiple model combinations for forecasting. Similarly, DWE-IL and OnlineTCN enhance prediction performance by incrementally training models on ELM and TCN network architectures, respectively. However, ensemble models are influenced by their base models and associated parameters, and when there are significant differences in data distribution, the variance in base model predictions can lower the overall prediction accuracy during the online prediction phase. ER and DER++ employ a replay mechanism, using a buffer to store a portion of the previous data and interleaving old samples while learning new ones. This, combined with Transformer’s ability to capture time series features, enhances the model’s long-term predictive capability in the online prediction phase. Compared to the classic Transformer model, all evaluation metrics have improved significantly. IL-ETransformer demonstrates superior performance across all evaluation metrics on both 000300.SH and 399905.SZ datasets. Its stronger feature extraction ability and long-term forecasting performance result in predictions that are markedly better than those of the baseline models.

**Fig 2:**
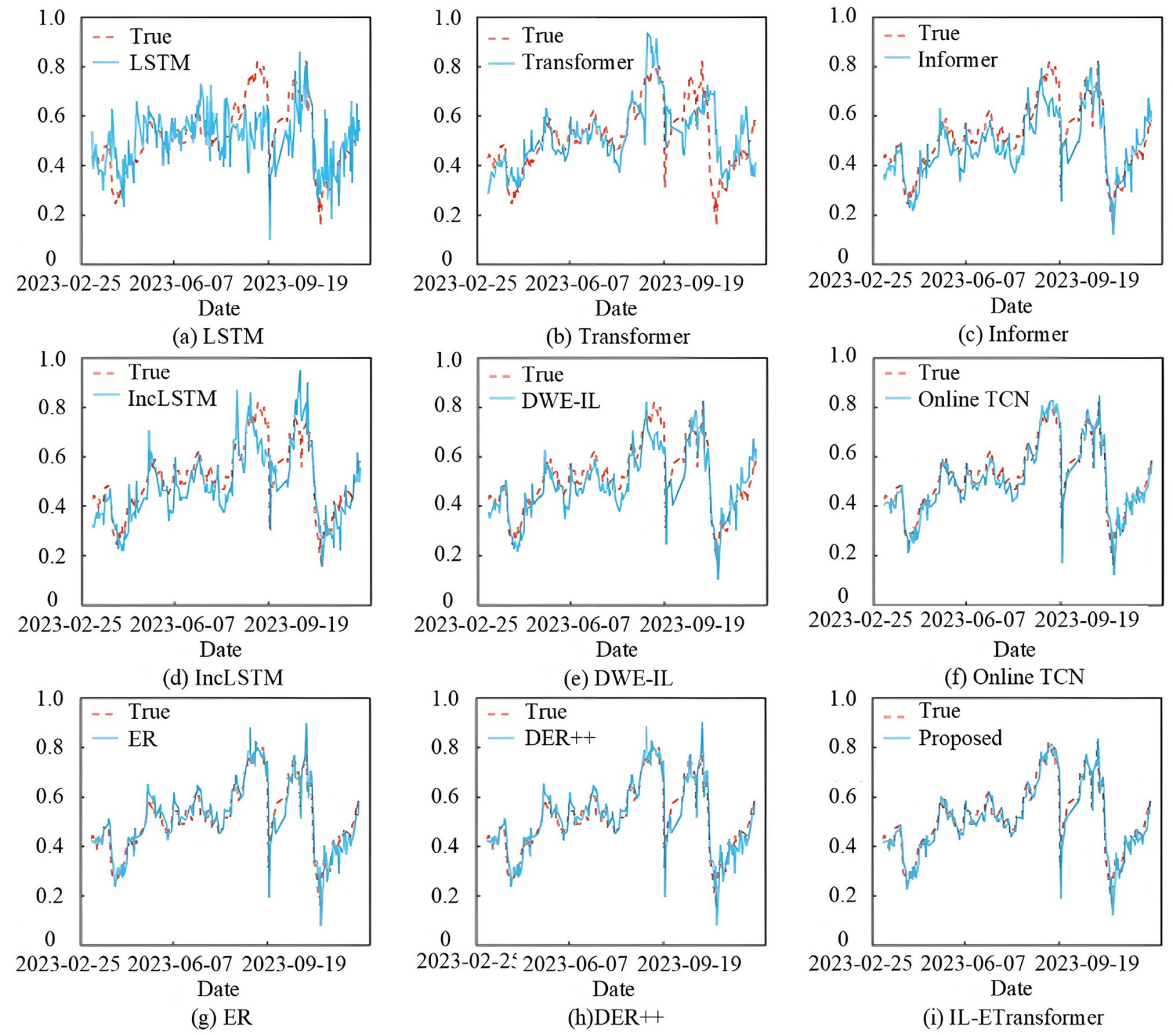
Prediction results of 000300.SH by each model.

**Fig 3:**
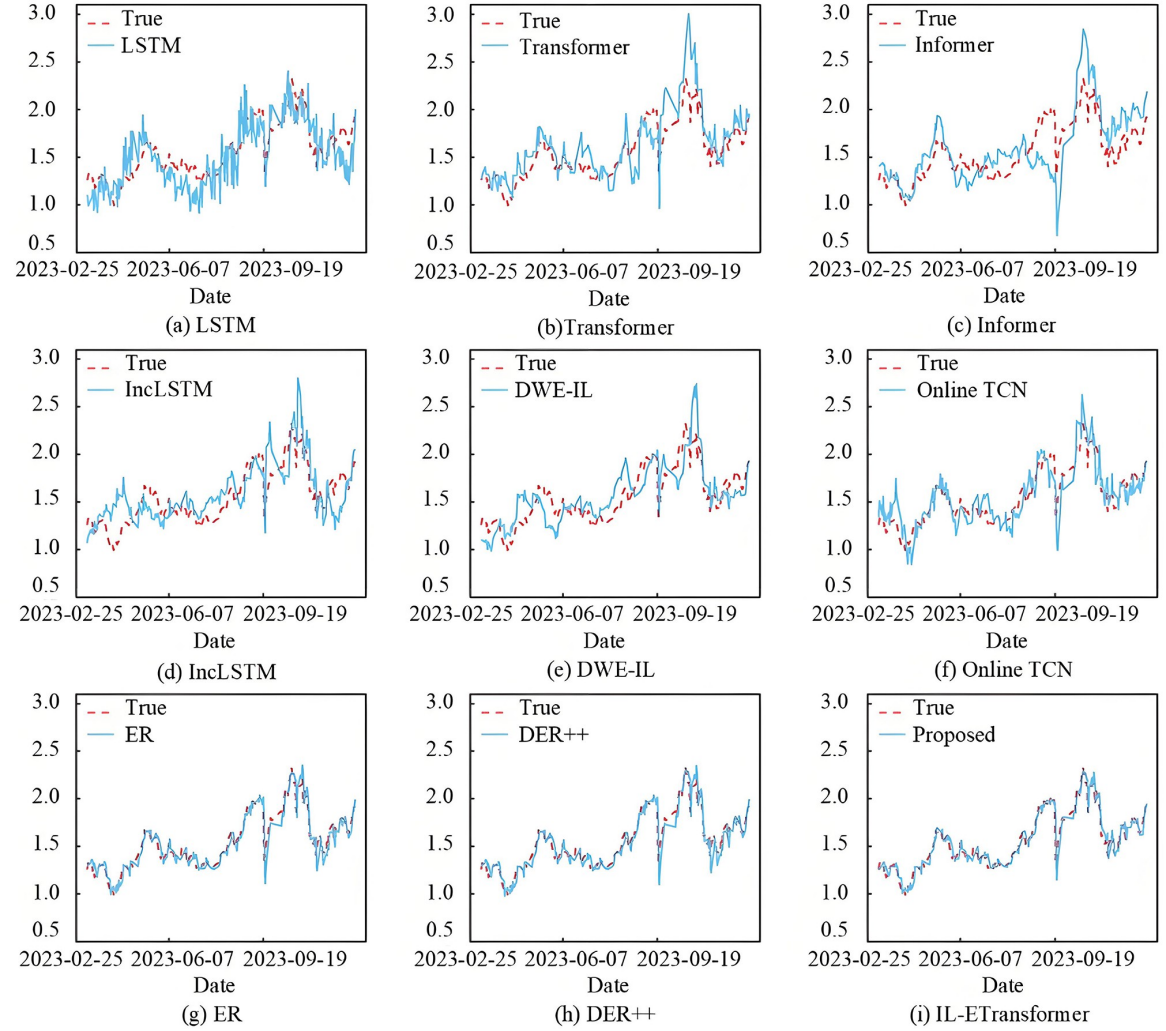
Prediction results of 399905.SZ by each model.

Figs 2 and 3 show the partial prediction results of the models during the online prediction phase for the 000300.SH and 399905.SZ, respectively. These figures display the predicted and actual closing prices, both of which have been standardized. In Figs 2 and 3, the predicted results from LSTM, Transformer, and Informer exhibit significant fluctuations in some intervals, even showing opposite trends to the actual data. This occurs because certain data distribution patterns did not appear during the warm-up phase, causing the static models to struggle in adapting to changes in new data trends. IncLSTM, DWE-IL, and OnlineTCN demonstrate some improvement due to their incremental learning capabilities, but their overall curve fitting remains suboptimal. Notably, these models face challenges in accurately predicting turning points when there are large fluctuations in the China Securities 500 Index. This indicates considerable room for improvement. On the other hand, the ER and DER++ methods, which utilize a replay mechanism with Transformer as the backbone network, provide predictions that are closer to the actual values and better capture the overall trend. In summary, IL-ETransformer achieves the best fitting performance, as the predicted and actual curves are the most closely aligned.

#### 4.3.2 Individual stock price prediction analysis

In real-world scenarios, the prices of individual stocks fluctuate randomly and are influenced by multiple factors, making them difficult to predict. To further verify the model’s accuracy, this study selects Ping An Bank (000001.SZ) from the 399905.SZ, and Chinese Media (600373.SH) and China National Medicines (600511.SH) from the 000300.SH for individual stock price prediction. Using IL-ETransformer and the baseline models, multiple experiments were conducted, and the average evaluation metrics during the online prediction phase are presented in Tables 3 to 5.

**Table 3:** Prediction results of 000001.SZ by each model.

Model	MAE	MSE	RMSE	MAPE
LSTM	0.416	0.764	0.729	1.746
Transformer	0.321	0.479	0.594	1.656
Informer	0.319	0.221	0.407	0.971
IncLSTM	0.327	0.218	0.416	0.981
DWE-IL	0.302	0.206	0.395	0.952
OnlineTCN	0.299	0.235	0.382	0.839
ER	0.190	0.076	0.222	0.555
DER++	0.146	0.047	0.169	0.528
IL-ETransformer	**0.132**	**0.043**	**0.157**	**0.526**

**Table 4:** Prediction results of 600373.SH by each model.

Model	MAE	MSE	RMSE	MAPE
LSTM	0.641	1.029	0.814	0.972
Transformer	0.552	0.863	0.735	0.945
Informer	0.527	0.842	0.706	0.795
IncLSTM	0.406	0.526	0.615	0.781
DWE-IL	0.356	0.452	0.523	0.618
OnlineTCN	0.289	0.235	0.338	0.439
ER	0.268	0.226	0.336	0.401
DER++	0.275	0.214	0.324	0.417
IL-ETransformer	0.253	0.172	0.298	0.372

**Table 5:** Prediction results of 600511.SH by each model.

Model	MAE	MSE	RMSE	MAPE
LSTM	0.375	0.409	0.496	1.643
Transformer	0.364	0.367	0.469	1.488
Informer	0.324	0.334	0.434	1.265
IncLSTM	0.327	0.256	0.417	1.368
DWE-IL	0.213	0.132	0.305	0.964
OnlineTCN	0.176	0.092	0.203	0.782
ER	0.164	0.108	0.249	1.046
DER++	0.163	0.082	0.193	0.773
IL-ETransformer	0.160	0.081	0.188	0.759

From the individual stock prediction results, it is evident that the IL-ETransformer outperforms the baseline models across all evaluation metrics. In incremental scenarios, it effectively predicts non-stationary and highly volatile stock price data, promptly learning from new data to dynamically adjust model parameters and better fit actual stock price trends. The IL-ETransformer exhibits strong generalization and robustness, providing important references for investors’ decision-making, thus enhancing investment returns.

Furthermore, compared to the stock index prediction results in Table 2, the IL-ETransformer model achieves the lowest MSE error for the Ping An Bank stock price series and the lowest MAPE for the Chinese Media stock price series. Although the MAPE for the China National Medicines price series is slightly higher, the other three evaluation metrics are significantly lower than those for the stock index series. Overall, the proposed model demonstrates superior prediction performance for individual stocks, effectively learning the trends and fluctuations in different stock price series.

### 4.4 Ablation experiment

To validate the prediction performance of the proposed IL-ETransformer model and the effectiveness of its functional modules, an ablation experiment is conducted using the 000300.SH dataset. This experiment involves removing different modules from the model to obtain variant models, followed by comparative analysis based on the evaluation metrics MAE and MSE. First, for the sustained attention module, it is replaced with the attention modules from Transformer and Informer, including LogSparse attention and ProbSparse attention. Second, the sustained normalization module is removed, leaving only layer normalization (LN). Lastly, the incremental training method is switched to EWC, ER, and DER++. During the process of replacing each module, all other components are kept constant. The experimental comparison results are presented in Table 6.

**Table 6:** IL-ETransformer ablation experiments.

Model	MAE	MSE
LogSparse attention	0.230	0.290
ProbSparse attention	0.232	0.288
IL-ETransformer (LN)	0.231	0.293
IL-ETransformer (ER)	0.245	0.315
IL-ETransformer (DER++)	0.237	0.305
IL-ETransformer (EWC)	0.228	0.285
IL-ETransformer	**0.225**	**0.274**

From Table 6, it is evident that the continuous attention mechanism of the IL-ETransformer demonstrates optimal performance, reducing MAE and MSE by 2.17% and 5.51%, respectively, compared to LogSparse attention. This validates that the proposed module is more suitable for online prediction scenarios and stream processing, effectively capturing the trend characteristics of new data. Furthermore, the removal of the continuous normalization mechanism results in increased error metrics, indicating that this module aids in data stabilization and helps the model continuously learn from new data. Regarding the replacement of incremental learning methods, EWC and TS-EWC show good prediction performance, with TS-EWC offering lower computational overhead and higher model update efficiency. As new data patterns emerge and the number of tasks the network parameters must learn increases, TS-EWC outperforms EWC. Overall, the components of the IL-ETransformer reduce the model’s computational overhead while enhancing its ability to capture temporal and feature dimensional associations in data, thereby effectively improving the overall performance of the online prediction framework.

## 5. Conclusion

To enhance the accuracy of stock price predictions, this study addresses the challenges faced by existing deep learning model structures in providing poor online prediction performance for non-stationary stock prices as prediction time increases and new data continually emerges. We propose the IL-ETransformer, an online prediction model based on incremental learning algorithms and the Transformer framework. This model introduces a continuous self-attention mechanism to explore the temporal dependencies among feature variables and employs a continuous normalization mechanism to stabilize data in incremental scenarios. Additionally, the use of the TS-EWC algorithm ensures the dynamic updating of the model, allowing for more effective predictions of new data. Experimental results demonstrate that the prediction performance and fitting degree of the IL-ETransformer significantly outperform classic deep learning and incremental learning methods, including LSTM, Transformer, Informer, IncLSTM, DWE-IL, OnlineTCN, ER, and DER++. Furthermore, various stock index and individual stock data selected for the experiments validate the model’s general applicability.

Given that financial time series are influenced by various factors such as political, social, economic, and psychological aspects, making accurate predictions remains challenging. This study only utilizes a subset of technical indicators for feature extraction and prediction. To further improve the model’s prediction accuracy, future work will consider integrating factors such as financial news, stock commentary sentiment, and investor sentiment. Additionally, optimizing the parameter settings of the IL-ETransformer model and incorporating interpretable optimization algorithms will provide more accurate and practical references for the financial sector.
